# Predicting Mortality in Intensive Care Unit Patients With Allergic Bronchopulmonary Aspergillosis (ABPA) Using an Interpretable Machine Learning Model: A Retrospective Cohort Study

**DOI:** 10.1155/carj/1423700

**Published:** 2026-04-15

**Authors:** Jing Zhang, Juntao Tang, Jinjuan Li, Xinghua Liu, Ye Sun, Peng Wang

**Affiliations:** ^1^ Intensive Care Unit, Yuebei People’s Hospital Affiliated to Shantou University School of Medicine, Shaoguan, Guangdong, China; ^2^ Neurosurgery, The Second Affiliated Hospital of Soochow University, Suzhou, Jiangsu, China, suda.edu.cn

**Keywords:** allergic bronchopulmonary aspergillosis (ABPA), machine learning, predictive model, XGBoost

## Abstract

**Background:**

Allergic bronchopulmonary aspergillosis (ABPA) is a hypersensitivity lung disease caused by *Aspergillus* infection, with severe cases often requiring admission to the intensive care unit (ICU). Early prediction of in‐hospital mortality in ICU ABPA patients is crucial for optimizing clinical decision‐making and resource allocation.

**Methods:**

This retrospective study collected clinical data from ICU patients diagnosed with ABPA at Yuebei People’s Hospital between January 2020 and July 2024. An in‐hospital mortality prediction model was developed using an explainable XGBoost machine learning algorithm. SHapley Additive Explanations (SHAP) was employed to interpret key predictive factors, and internal validation was conducted to assess model performance.

**Results:**

A total of 82 ICU ABPA patients were included, with mortality rates of 46.3% (26/57) in the training set and 48% (12/25) in the validation set. The XGBoost model demonstrated excellent predictive performance, achieving areas under the receiver operating characteristic (ROC) curve (AUC) of 0.995 (95% CI: 0.903–1.000) in the training set and 0.881 (95% CI: 0.846–0.909) in the validation set. SHAP analysis identified key predictors of mortality, including BMI, peak procalcitonin level, peak eosinophil count, age, asthma history, peak leukocyte count, and lowest platelet count.

**Conclusion:**

The XGBoost model effectively predicts in‐hospital mortality in ICU ABPA patients and provides interpretable results using SHAP analysis. Although the model performed well in internal validation, external validation is needed to enhance its generalizability. Future multicenter studies and integration of dynamic biomarkers are recommended to optimize predictive accuracy and support individualized clinical decision‐making.

## 1. Introduction

Allergic bronchopulmonary aspergillosis (ABPA) is a hypersensitivity lung disease caused by a complex immune reaction triggered by *Aspergillus* colonization in the airways [1, 2]. ABPA typically occurs in patients with preexisting respiratory conditions, such as asthma and cystic fibrosis (CF), and it is characterized by difficult‐to‐control asthma‐like symptoms and recurrent pulmonary infiltrates, with or without bronchiectasis. With increasing awareness among clinicians, ABPA has also been identified as a secondary condition in patients with bronchiectasis, chronic obstructive pulmonary disease (COPD), and other pulmonary disorders [3, 4]. The prevalence of ABPA in the general population remains unclear; however, it has been reported to range from 2% to 32% in asthma patients [4] and from 3% to 25% in CF patients [5].

The prognosis of ABPA largely depends on early diagnosis and timely treatment. The natural course of ABPA is variable, with recurrent exacerbations and remissions being its hallmark. Long‐term recurrence can lead to irreversible lung damage, including bronchiectasis, pulmonary fibrosis, and even progression to chronic pulmonary heart disease or respiratory failure. Serum total IgE levels are commonly used as a biomarker for monitoring ABPA disease activity [6]. High‐attenuation mucus (HAM) impaction and the severity of central bronchiectasis have been identified as independent risk factors for ABPA relapse [7, 8].

Critically ill patients in the intensive care unit (ICU) require specialized care and multidisciplinary support [9]. Although the ICU plays a crucial role in sustaining patients’ lives, it also poses challenges, such as workforce shortages, limited medical resources, and significant financial burdens [10]. Therefore, early identification of hospital mortality risk in ICU patients is essential, as it may facilitate appropriate treatment strategies and support clinical decision‐making [11].

In recent years, artificial intelligence has been widely employed to identify early warning predictors for various diseases. Given the inherent strength of machine learning algorithms in capturing nonlinear relationships, an increasing number of researchers advocate for the use of machine learning–based predictive models to support appropriate patient treatment, rather than relying solely on traditional disease severity scoring systems, such as the Sequential Organ Failure Assessment (SOFA), Acute Physiology and Chronic Health Evaluation II (APACHE II), or Simplified Acute Physiology Score II (SAPS II) [12–14]. Although numerous predictive models have demonstrated promising performance in research settings, their clinical application remains limited, and there is still a lack of evidence supporting interpretable risk prediction models that contribute to disease prognosis [15–18].

The use of an explainable machine learning model based on Extreme Gradient Boosting (XGBoost) to predict mortality in ICU patients with ABPA and to perform internal validation represents an emerging research direction. By analyzing clinical data, the XGBoost model can effectively predict mortality in ABPA patients while providing interpretable predictions. Internal validation, achieved through methods, such as cross‐validation, ensures the stability and reliability of the model. This approach not only assists clinicians in better assessing patient prognosis but also provides valuable insights for developing personalized treatment strategies.

The objective of this study was to utilize existing clinical data and patient characteristics from ICU admission to construct and interpret an XGBoost model for ABPA prognosis. The SHapley Additive Explanations (SHAP) method is employed to explain the model’s predictions and explore prognostic factors associated with ABPA.

## 2. Methods

### 2.1. Data Source

This retrospective study collected clinical data from patients diagnosed with ABPA at Yuebei People’s Hospital between January 2020 and July 2024. Because ABPA is an immune‐mediated hypersensitivity disorder rather than a primary fungal infection, the final diagnosis was adjudicated in accordance with the 2017 Expert Consensus on the Diagnosis and Treatment of ABPA established by the Asthma Group of the Chinese Thoracic Society, integrating clinical manifestations, immunologic evidence, laboratory findings, and radiologic characteristics. Metagenomic next‐generation sequencing (mNGS) of sputum was used only as supportive microbiological evidence to indicate *Aspergillus*‐related exposure/colonization signals and was not considered diagnostic on its own. The dataset comprehensively recorded demographic information, vital signs, diagnostic details, and treatment information for all patients.

The inclusion criteria were based on the **2017 Expert Consensus on the Diagnosis and Treatment of Allergic Bronchopulmonary Aspergillosis** established by the Asthma Group of the Chinese Thoracic Society. Patients were included if they met the following criteria:

1. Associated diseases
 Presence of asthma or other underlying respiratory conditions, such as bronchiectasis, COPD, or pulmonary CF.

2. Essential Criteria:
  1. *Aspergillus fumigatus*–specific IgE > 0.35 kUA/L or a positive immediate skin test reaction to *Aspergillus fumigatus.*
  2. Elevated serum total IgE levels (> 1000 IU/mL).

3. Additional Criteria:
  1. Blood eosinophil count > 0.5 × 10^9^/L.
  2. Pulmonary imaging findings consistent with ABPA, including but not limited to pulmonary consolidation, nodules, toothpaste sign, finger‐in‐glove sign, transient migratory opacities, or persistent changes, such as bronchiectasis and pleuroparenchymal fibrosis.
  3. Positive serum *Aspergillus*‐specific IgG antibodies or precipitin test.

A diagnosis of ABPA required meeting criterion **1**, criterion **2**, and at least two conditions from criterion **3**. Patients with serum total IgE levels < 1000 IU/mL could still be diagnosed with ABPA if all other criteria were met. Additionally, only patients aged 18 years or older were included in the study.

The exclusion criteria were as follows:

1. Presence of malignancy or severe organ dysfunction (e.g., cardiac, cerebral, or renal failure).
2. Pregnant or lactating women.
3. Incomplete medical records or insufficient diagnostic data.

## 3. Ethical Considerations

This study was approved by the Ethics Committee of Yuebei People’s Hospital. As all protected health information was anonymized and the study was retrospective in nature, the requirement for informed consent from patients and their families was waived.

### 3.1. Predictor Variables and Explainable Machine Learning Tools

The outcome variable in this study was the probability of in‐hospital mortality, defined based on the patient’s status at discharge. Model interpretability was enhanced using SHAP, which quantifies the individual contribution of each feature to the prediction outcome [19]. SHAP values indicate the extent to which each predictor variable influences the target variable, either positively or negatively. Additionally, each individual observation in the dataset can be explained by a specific set of SHAP values.

### 3.2. Statistical Analysis

All statistical analyses and computations were performed using R software (Version 3.8.0). Categorical variables were expressed as counts and percentages, and differences between groups were compared using the chi‐square (*χ* ^2^) test or Fisher’s exact test (when the expected frequency was < 10). Continuous variables were reported as medians with interquartile ranges (IQR) and were compared between groups using the Wilcoxon rank‐sum test.

The predictive model was developed using the XGBoost machine learning algorithm. Model performance was evaluated by calculating the area under the receiver operating characteristic (ROC) curve (AUC). Additionally, accuracy, sensitivity, positive predictive value (PPV), and negative predictive value (NPV) were computed. To assess the clinical utility of the model, decision curve analysis (DCA) was conducted, which quantifies net benefit across different probability thresholds for decision‐making [20].

SHAP values were calculated using the R language implementation, enabling interpretation of individual feature contributions to the model predictions, which is specifically optimized for tree‐based models, such as XGBoost. This approach enables calculation of consistent, locally accurate feature attribution values for each patient’s mortality prediction.

## 4. Results

### 4.1. Patient Characteristics

**FIGURE 1:**
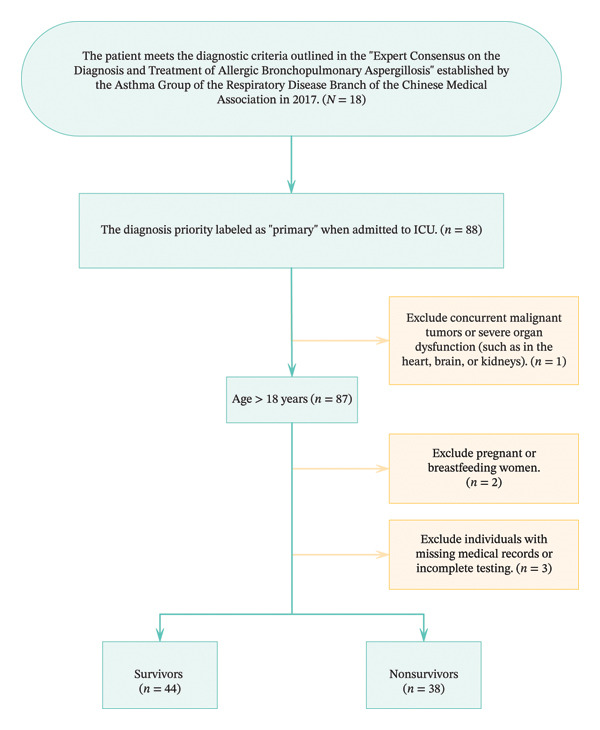
Flowchart of patient selection.

A total of 82 adult patients diagnosed with ABPA in the ICU were included in the final cohort of this study. The patient screening process is illustrated in Figure 1. The dataset was split into a training set (70%) and a validation set (30%) using stratified random sampling based on the outcome variable (in‐hospital mortality) to ensure balanced distribution of the event across the subsets. The dataset was randomly divided into two subsets: 70% (*n* = 57) of the data was used for model training, while 30% (*n* = 25) was used for model validation. A total of 24 potential predictive factors were identified and used for model development.

Patients in the nonsurvivor group were older than those in the survivor group (*p* = 0.04). The in‐hospital mortality rate was 46.3% (26/57) in the training dataset and 48% (12/25) in the testing dataset. Table 1 presents a comparison of predictor variables between survivors and nonsurvivors during hospitalization.

**TABLE 1:** All predictor variables for patients with ABPA.

Characteristic	All‐cause mortality	Survival	*p* value
Total N	38	44	
Baseline variables and in‐hospital factors Age (years)	68.82 ± 11.69	74.07 ± 11.12	0.040
BMI (kg/m^2^)	23.89 ± 2.80	25.05 ± 3.04	0.078
Shock	1.00 (0.00–1.00)	0.00 (0.00–1.00)	0.102
Peak leukocyte count (10^9^/L)	17.91 (14.75–32.52)	15.54 (11.02–22.93)	0.045
Peak eosinophil count (10^9^/L)	0.19 (0.01–0.54)	0.33 (0.15–0.56)	0.129
Lowest hemoglobin concentration (g/L)	64.00 (52.25–77.00)	71.00 (58.00–82.50)	0.154
Lowest platelet count (10^9^/L)	49.50 (32.00–102.50)	93.50 (26.00–147.75)	0.076
Peak procalcitonin level (ng/mL)	11.11 (1.92–28.92)	1.97 (0.88–6.90)	< 0.001
Peak C‐reactive protein (CRP) level (mg/dL)	29.43 (10.78–33.06)	15.29 (10.13–27.54)	0.102
Peak blood glucose level (mmol/L)	13.20 (12.03–17.88)	12.89 (9.90–18.50)	0.802
Gender			0.429
Male	32 (84.21%)	34 (77.27%)	
Female	6 (15.79%)	10 (22.73%)	
Comorbidities: asthma history			0.054
N	25 (65.79%)	37 (84.09%)	
Y	13 (34.21%)	7 (15.91%)	
History of corticosteroid use			0.005
N	28 (73.68%)	42 (95.45%)	
Y	10 (26.32%)	2 (4.55%)	
Smoking history			0.852
N	35 (92.11%)	41 (93.18%)	
Y	3 (7.89%)	3 (6.82%)	
Allergic rhinitis			0.057
N	38 (100.00%)	40 (90.91%)	
Y	0 (0.00%)	4 (9.09%)	
Food and drug allergies			0.203
N	36 (94.74%)	38 (86.36%)	
Y	2 (5.26%)	6 (13.64%)	
Diabetes mellitus			0.356
N	27 (71.05%)	27 (61.36%)	
Y	11 (28.95%)	17 (38.64%)	
Concurrent bacterial infection			0.576
N	17 (44.74%)	17 (38.64%)	
Y	21 (55.26%)	27 (61.36%)	
Concurrent viral infection			0.375
N	28 (73.68%)	36 (81.82%)	
Y	10 (26.32%)	8 (18.18%)	
Renal insufficiency or failure			< 0.001
N	6 (15.79%)	24 (54.55%)	
Y	32 (84.21%)	20 (45.45%)	
Hepatic insufficiency or failure			0.165
N	28 (73.68%)	26 (59.09%)	
Y	10 (26.32%)	18 (40.91%)	
Pulmonary fibrosis			0.010
N	26 (68.42%)	40 (90.91%)	
Y	12 (31.58%)	4 (9.09%)	
Pulmonary cavities or bronchiectasis			0.017
N	15 (39.47%)	29 (65.91%)	
Y	23 (60.53%)	15 (34.09%)	
Pulmonary infiltration			0.231
N	14 (36.84%)	22 (50.00%)	
Y	24 (63.16%)	22 (50.00%)	

*Note:* The variables age and BMI were compared using the *t-test*. The variables peak leukocyte count, peak eosinophil count, lowest hemoglobin concentration, lowest platelet count, peak procalcitonin level, peak C‐reactive protein (CRP) level, and peak blood glucose level were analyzed using the *Mann–Whitney U test*. Categorical variables, including shock, gender, asthma history, history of corticosteroid use, smoking history, allergic rhinitis, food and drug allergies, diabetes mellitus, concurrent bacterial infection, concurrent viral infection, renal insufficiency or failure, hepatic insufficiency or failure, pulmonary fibrosis, pulmonary cavities or bronchiectasis, and pulmonary infiltration, were analyzed using the *chi-square test*.

### 4.2. Model Development and Evaluation

**FIGURE 2:**
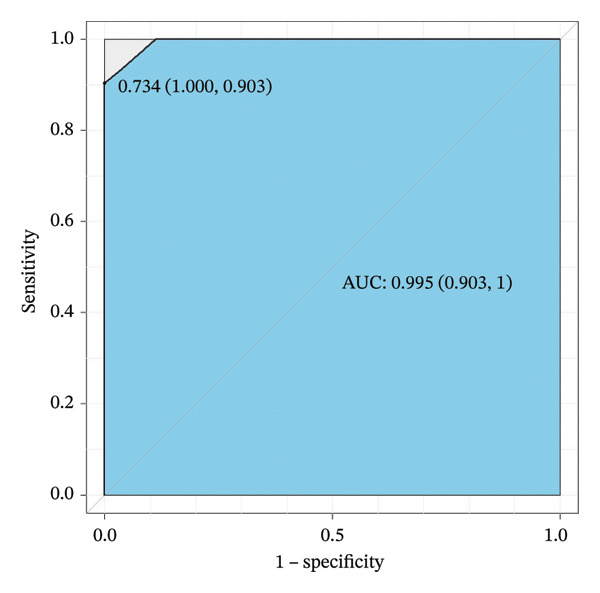
Receiver operating characteristic (ROC) curve of the XGBoost model in the training cohort of patients with ABPA.

An XGBoost model was developed using the training dataset, and its ROC curve for the test dataset is shown in Figure 2, with an AUC of 0.995 (95% CI: 0.903–1.000).

**FIGURE 3:**
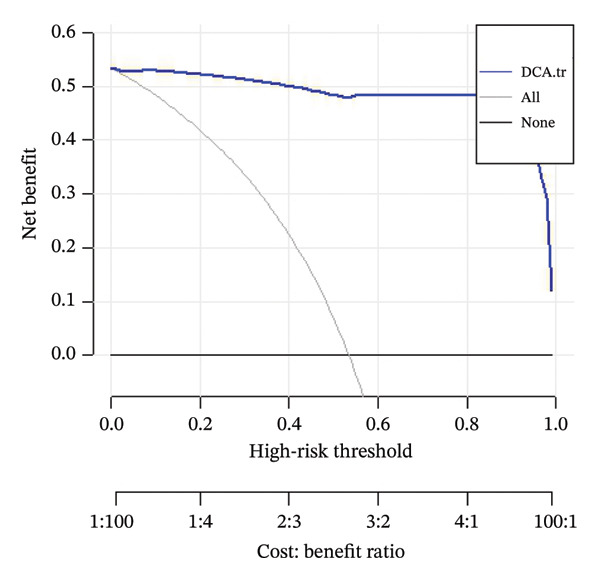
Decision curve analysis (DCA) of the predictive model in the training cohort for in‐hospital mortality among ICU patients with ABPA. The *y*‐axis indicates net benefit, and the *x*‐axis shows the high‐risk threshold (probability threshold), with the lower axis displaying the corresponding cost:benefit ratio. The blue curve (“DCA.tr”) represents the model’s net benefit across thresholds, the gray curve (“All”) represents the net benefit of treating all patients, and the black line (“none”) indicates the net benefit if no patients are treated. The DCA demonstrates that the model provides a higher net benefit across a wide range of clinically relevant thresholds compared to “treat all” and “treat none” strategies.

DCA was performed on the test dataset to compare the net benefit of the optimal model against alternative clinical decision‐making strategies. Clinical net benefit was defined as the minimum probability of disease occurrence at which further intervention would be warranted [21]. The DCA curve quantifies net benefit across different probability thresholds, as shown in Figure 3.

**FIGURE 4:**
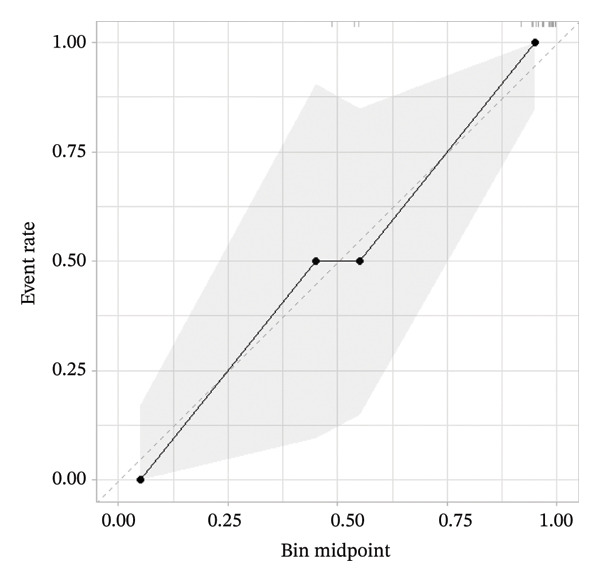
Calibration curve analysis of the XGBoost model in the training cohort of patients with ABPA.

A calibration curve illustrating the discrepancy between predicted and actual mortality is presented in Figure 4. Due to the heterogeneity of the study population, machine learning–based treatment strategies outperformed the default strategies of treating all or treating none of the patients.

**FIGURE 5:**
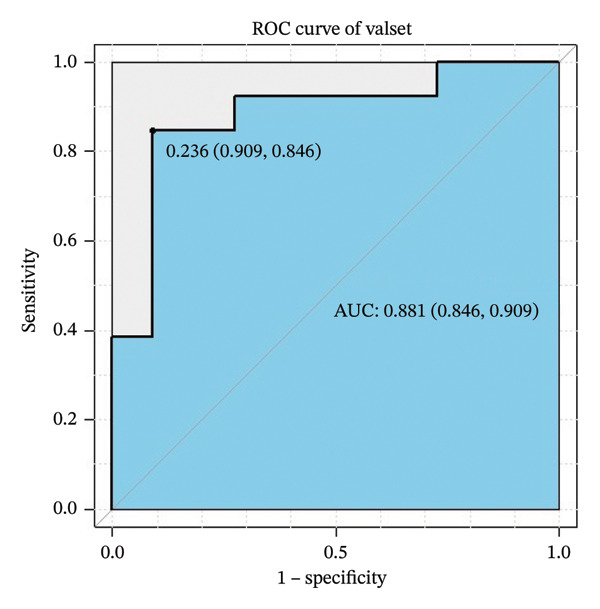
Receiver operating characteristic (ROC) curve of the XGBoost model in the validation cohort of patients with ABPA.

**FIGURE 6:**
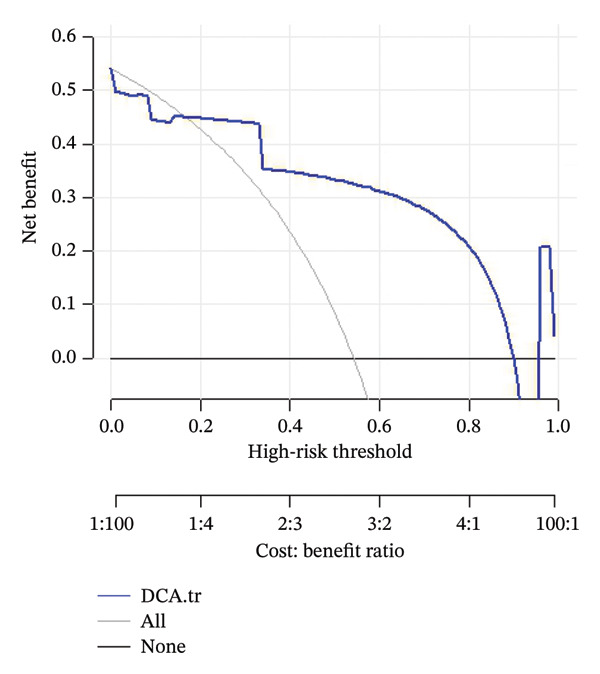
Decision curve analysis (DCA) of the predictive model in the validation cohort for in‐hospital mortality among ICU patients with ABPA. The *y*‐axis represents net benefit, while the *x*‐axis indicates the high‐risk threshold (probability threshold). The lower axis shows the corresponding cost: benefit ratio for each threshold. The blue curve (“DCA”) shows the net benefit of the predictive model in the validation cohort across different thresholds. The gray curve (“all”) represents the net benefit of treating all patients, and the black line (“none”) represents the net benefit of treating no patients. The DCA demonstrates that the predictive model provides a higher net benefit across a clinically relevant range of thresholds compared to “treat all” and “treat none” strategies.

**FIGURE 7:**
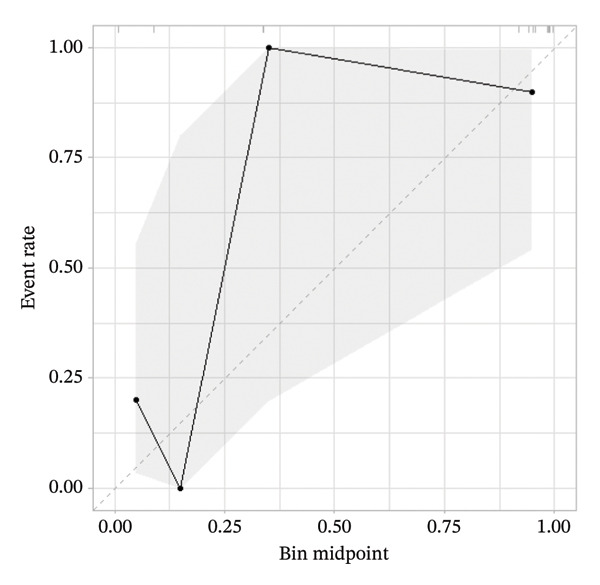
Calibration curve analysis of the XGBoost model in the validation cohort of patients with ABPA.

For the validation dataset, the ROC curve is shown in Figure 5, with an AUC of 0.881 (95% CI: 0.846–0.909). Additionally, DCA (Figure 6) and calibration curve analysis (Figure 7) were conducted on the validation dataset, demonstrating excellent predictive performance of the model.

### 4.3. Interpretation of the XGBoost Model Using the SHAP Method

**FIGURE 8:**
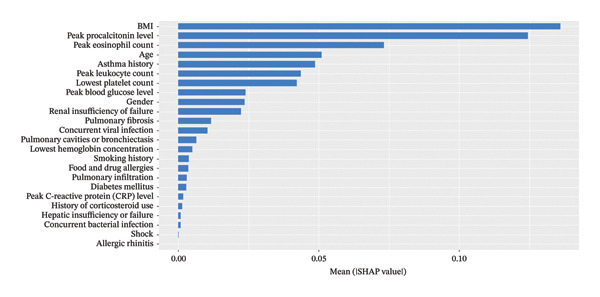
The weights of variable importance.

The SHAP algorithm was employed to assess the importance of each predictor variable in the XGBoost model’s predictions. The variable importance plot ranks the most significant features in descending order (Figure 8). Among all predictors, **BMI** had the strongest predictive value, followed by **peak procalcitonin level, peak eosinophil count, age, asthma history, peak leukocyte count,** and **lowest platelet count**.

**FIGURE 9:**
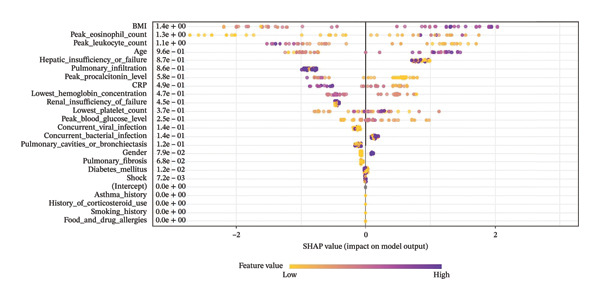
The SHapley additive exPlanations (SHAP) values.

Additionally, to determine the direction of the relationship between predictors and the outcome, SHAP values were used to identify mortality risk factors. As shown in Figure 9, the horizontal position indicates whether a given feature’s effect is associated with a higher or lower mortality prediction, while the color represents whether the variable value is high (red) or low (blue) for a specific observation. The results indicate that an **increase in BMI positively influences mortality prediction**, shifting it toward a higher probability of death, whereas **an increase in age negatively influences mortality prediction**, shifting it toward survival.

### 4.4. SHAP Force Plot

**FIGURE 10:**
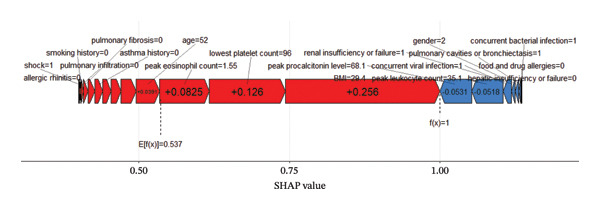
Individualized SHapley additive exPlanations (SHAP) force plot interpretation for risk stratification in allergic bronchopulmonary aspergillosis (ABPA) patients.

Figure 10 presents the individual force plot for a specific patient. The SHAP values illustrate the predictive features relevant to that patient and the contribution of each feature to the mortality prediction. The **bold number** represents the probability prediction value (*f(x)*), while the **baseline value** corresponds to the model’s prediction without any input data. *f(x)* represents the log‐odds ratio for each observation.

In the force plot, red features (on the left) represent variables that increase the model’s predicted risk of mortality for that individual patient, while blue features (on the right) represent variables that decrease the prediction. The length of the arrows visually represents the magnitude of each feature’s influence on the prediction—the longer the arrow, the greater the impact.

## 5. Discussion

In this study, we developed an XGBoost machine learning model based on clinical data from the ICU to predict in‐hospital mortality in patients with ABPA. The model’s performance was evaluated through internal validation. Model performance was assessed using internal validation. Although the model achieved very high discrimination in the training cohort (AUC ≈ 0.995), the relatively small sample size (*n* = 82) in combination with multiple predictors raises concerns regarding potential overfitting and optimistic performance estimates. Therefore, the present findings should be interpreted as exploratory. DCA suggested a potential net clinical benefit within a predicted probability threshold range of 10%–20%, indicating that the model may serve as an adjunctive tool for early ICU risk stratification and hypothesis generation when used alongside clinical judgment, rather than as a standalone system for direct clinical decision‐making. Using SHAP for model interpretation, we identified key features associated with mortality risk, including BMI, peak procalcitonin level, peak eosinophil count, age, asthma history, peak leukocyte count, and lowest platelet count. These results improve the transparency and interpretability of the model and provide clinically relevant signals that warrant further external validation and prospective evaluation to confirm generalizability and clinical utility.

## 6. Clinical Significance of Key Variables

Through an in‐depth analysis of the model’s feature contributions using the SHAP method, we identified **seven key clinical predictors** associated with mortality in ABPA patients. Among them, **BMI** was the most significant predictor of mortality. Other important variables, such as **peak procalcitonin level, peak eosinophil count, age, asthma history, peak leukocyte count,** and **lowest platelet count**, also played critical roles in patient prognosis [22].

**BMI**, as an indicator of metabolic status and systemic inflammation, may influence patient outcomes by exacerbating systemic inflammatory responses. Previous studies have also linked **BMI** to the severity of pulmonary diseases [23–26]. An **elevated peak procalcitonin level** is typically indicative of severe infections, and its abnormal levels in ABPA may signal a poor prognosis [26]. Additionally, an **increase in peak eosinophil count** is associated with more severe airway inflammation, which is closely related to the immunopathological mechanisms of ABPA [27].

**Age** is a key factor affecting immune function and may contribute to disease progression in ABPA. Studies have shown that older patients are more likely to experience adverse clinical outcomes [27]. Interestingly, in our cohort, SHAP analysis suggested a negative correlation between age and mortality, which may be due to selection bias where younger ICU patients had more severe disease presentations, warranting further validation in larger cohorts. A **history of asthma** is closely linked to the pathogenesis of ABPA, as persistent airway inflammation and structural changes can increase disease complexity. Furthermore, an **elevated peak leukocyte count** often indicates systemic inflammation or infection, which may be associated with a higher risk of mortality in severe cases [28]. A **decrease in the lowest platelet count** may reflect endothelial damage or coagulation dysfunction, which is also a marker of poor prognosis in critically ill ABPA patients [29].

In this study, we developed a mortality risk prediction model for patients with ABPA in the ICU setting, utilizing XGBoost combined with SHAP for model interpretability. This work helps to address a gap in the current literature by focusing on a rare disease (ABPA) within the unique context of critical care.

Over the past 5 years, relevant studies have shown that while machine learning has been widely applied for outcome prediction among critically ill ICU patients (such as those with pneumonia or invasive fungal infections), research specifically targeting ABPA patients—particularly those in the ICU—remains extremely limited. For example, Wang et al. developed a machine learning model for identifying invasive pulmonary aspergillosis in non‐neutropenic patients, which included nine ABPA cases, suggesting that machine learning can aid diagnosis and risk stratification in fungal‐related diseases; however, its application was limited to specific subgroups and did not focus on mortality prediction in ICU patients with ABPA [30]. Similarly, Du et al. proposed a prognostic model combining lasso regression and machine learning for non‐neutropenic pulmonary aspergillosis but did not explicitly include ABPA nor target ICU patients [31]. In contrast, Jeon et al. and Wang et al. have conducted machine learning–based mortality prediction studies in ICU patients with pneumonia, using methods, such as random forests and XGBoost, demonstrating significant improvements over traditional scoring systems [32, 33].

Additionally, Chia et al. explored the application of interpretable machine learning for mortality prediction in the ICU, emphasizing the importance of model transparency for clinical acceptance, which aligns closely with our use of SHAP in this study [34].

Taken together, compared to existing literature, our study expands and contributes to the field in several key aspects:

1. Unique Study Population: This is the first study to develop a mortality risk prediction model specifically for ICU patients with ABPA, helping to fill an existing research gap.
2. Methodological Innovation: By applying XGBoost in combination with SHAP, we aim to enhance predictive performance while ensuring model interpretability, providing a practical tool for clinical application.
3. Value for Small Sample, Rare Disease Modeling: Our approach offers experience and insight into developing predictive models for rare diseases under low‐sample conditions, which may help advance research in similar clinical areas.

We hope these contributions will provide a useful reference for future studies in the field and support the development of effective, interpretable machine learning applications in critical care for rare disease populations.

In summary, these key clinical features provide a solid basis for model interpretation and offer valuable reference points for risk assessment in clinical interventions. The ability of the **XGBoost** model to integrate these variables through nonlinear relationships highlights the advantages of machine learning in capturing complex clinical interactions. It is important to note that the SHAP force plot illustrates the contribution of each feature to an individual prediction and does not necessarily reflect general clinical associations.

### 6.1. Model Limitations and Challenges in Clinical Translation

Although the model demonstrated robustness through internal validation, several limitations should be considered [35]. First, the mortality rate among ABPA patients in the final cohort was relatively low (46.34%). At a threshold corresponding to 80% specificity, the model’s PPV was 0.20, likely reflecting sample heterogeneity and moderate event prevalence. This result may be influenced by sample heterogeneity within the single‐center dataset and the relative rarity of severe ABPA cases. Additionally, the **lack of an external validation cohort** may limit the generalizability of the model, particularly in patients with immunosuppression or other pulmonary comorbidities.

Currently, this model is better suited for **early ICU risk stratification or screening** rather than direct clinical decision‐making. **Specifically, the model is intended to be applied early after ICU admission (e.g., within the first 24 h, or once routine laboratory results become available)** to generate a predicted mortality risk that may help **prioritize closer monitoring, prompt early re-assessment, and support triage of clinical attention and resources**. **Importantly, the model output should be used only as an adjunct to clinician judgment and should not be treated as a standalone decision-support system or used in isolation to mandate specific therapies.** Future prospective studies should evaluate the actual benefit and cost‐effectiveness of **workflow-integrated, risk-guided early management strategies** (e.g., **intensified monitoring, standardized re-evaluation, and guideline-concordant consideration of antifungal indication and/or immunomodulation**) for high‐risk patients.

A preprint version of this work is available on Research Square (https://doi.org/10.21203/rs.3.rs-7822617/v1) [36]. The present manuscript represents a revised and expanded version of the preprint. In particular, we have (i) corrected the diagnostic criteria and strengthened the supporting rationale by adding the relevant references, (ii) refined and completed selected figures and associated legends to improve clarity and completeness, and (iii) incorporated additional revisions that enhance the overall rigor and quality of reporting, informed by feedback received during a prior peer‐reviewed process. Collectively, these updates improve methodological transparency and the robustness of the presentation, while the principal findings and overarching conclusions remain broadly consistent with those reported in the preprint. The preprint has not been formally published in any peer‐reviewed journal.

## 7. Conclusion

This study demonstrated that the **XGBoost** model can effectively predict mortality risk in **ABPA** patients and, through interpretability analysis, reveal key pathophysiological mechanisms. However, **clinical translation of the model requires addressing limitations, such as low PPV and the absence of external validation**. Future research should incorporate **multicenter cohorts** and **dynamic biomarkers** (e.g., serial IgE monitoring or radiomic features of pulmonary imaging) to optimize predictive accuracy and support **personalized treatment strategies**.

## Author Contributions

Peng Wang, Ye Sun, and Jing Zhang designed the work. Juntao Tang, Jinjuan Li, and Xinghua Liu recorded and summarized the patient’s features. Peng Wang and Jing Zhang analyzed datasets. Jing Zhang wrote this paper.

## Funding

The authors have nothing to report.

## Disclosure

A preprint version of this manuscript has been posted on Research Square (https://www.researchsquare.com/article/rs-7822617/v1) [36]. The current submission represents a revised and expanded version of the preprint. The preprint has been cited in the reference list to ensure transparency and compliance with journal policies. All authors read and approved the final manuscript.

## Ethics Statement

This study was reviewed and approved by the Ethics Committee of Yuebei People’s Hospital (Ethics Approval Number: YBSKY‐2025‐029‐001). The committee granted a waiver of informed consent for this research in accordance with national regulations and institutional ethical guidelines. The waiver was justified based on the retrospective and anonymized nature of the data analyzed, which posed no foreseeable risks to participants’ privacy or rights. All personal identifiers were removed from the dataset before analysis to ensure confidentiality. The study strictly adhered to the principles outlined in the Declaration of Helsinki and relevant Chinese ethical standards for biomedical research involving human subjects.

## Conflicts of Interest

The authors declare no conflicts of interest.

## Data Availability

The datasets used and/or analyzed during this study are available from the corresponding author upon reasonable request.
